# Analysis of ovarian tumor pathology by Fourier Transform Infrared Spectroscopy

**DOI:** 10.1186/1757-2215-3-27

**Published:** 2010-12-21

**Authors:** Ranjana Mehrotra, Gunjan Tyagi, Deepak K Jangir, Ramesh Dawar, Noopur Gupta

**Affiliations:** 1 Optical Radiation Standards, National Physical Laboratory, (Council of Scientific and Industrial Research, New Delhi), Dr K S Krishnan Marg, New Delhi 110012, India; 2 Department of Pathology, Dharamshila Cancer Hospital and Research Centre, Vasundhara Enclave, Delhi 110096, India

## Abstract

**Background:**

Ovarian cancer is the second most common cancer among women and the leading cause of death among gynecologic malignancies. In recent years, infrared (IR) spectroscopy has gained attention as a simple and inexpensive method for the biomedical study of several diseases. In the present study infrared spectra of normal and malignant ovarian tissues were recorded in the 650 cm^-1 ^to 4000 cm^-1 ^region.

**Methods:**

Post surgical tissue samples were taken from the normal and tumor sections of the tissue. Fourier Transform Infrared (FTIR) data on twelve cases of ovarian cancer with different grades of malignancy from patients of different age groups were analyzed.

**Results:**

Significant spectral differences between the normal and the ovarian cancerous tissues were observed. In particular changes in frequency and intensity in the spectral region of protein, nucleic acid and lipid vibrational modes were observed. It was evident that the sample-to-sample or patient-to-patient variations were small and the spectral differences between normal and diseased tissues were reproducible.

**Conclusion:**

The measured spectroscopic features, which are the spectroscopic fingerprints of the tissues, provided the important differentiating information about the malignant and normal tissues. The findings of this study demonstrate the possible use of infrared spectroscopy in differentiating normal and malignant ovarian tissues.

## Background

Ovarian cancer is one of the leading causes of cancer-related deaths among women worldwide. In India, the Indian Council of Medical Research reports the incidence rate of ovarian cancer as 4.2 per 100,000 women [1]. A woman has a lifetime risk of ovarian cancer of around 1.5%, which makes it the second most common gynecologic malignancy [2]. Ovarian cancer usually occurs in women over the age of 50 years, but it can also affect younger women. Two types of ovarian cancers are found based on the cell types. Epithelial ovarian cancer, which starts in the surface layer covering the ovary and constitutes 80 to 90% of all tumours of the ovary. Germ line ovarian tumors which are derived from the germ cells of the ovary and occur much less frequently. The survival rate of ovarian cancer patient depends upon the stage at which the cancer is diagnosed. But ovarian cancer is hard to detect early, as early stage is generally asymptomatic. More than 75% of ovarian cancers are diagnosed with late stage disease. Patients would have a significantly-improved survival if their cancer could be detected while still limited to the ovary [3].

There is a widespread interest in developing screening methods for early ovarian cancer detection because of the high mortality associated with late stage disease. Presently, the test available for screening ovarian cancer patients focus on two areas. One is the assessment of certain biomarkers in the blood. The second area is of producing detailed images of ovaries through various imaging techniques. The most commonly used blood serum biomarker is Cancer Antigen 125 (CA-125) [4]. Specificity is not achieved by this test as other types of cancer can raise the CA-125 levels such as breast, endometrium, gastrointestinal tract, and lung cancer. CA-125 testing is also not effective in women who are pre-menopausal because the CA-125 level fluctuates during the menstrual cycle [5].

On the imaging area of study several imaging techniques have been employed such as Computed Tomography (CT), Magnetic Resonance Imaging (MRI) and Ultrasound Imaging. Studies have shown that ultrasound gives a poor accuracy in detecting early stage disease [6]. A much more accurate ultrasound imaging screening test is the Trans Vaginal Ultrasonography (TVS) which gives impressive results, however it is inefficient in distinguishing between benign and malignant masses. The only way to diagnose ovarian cancer with certainty is an exploratory operation. But it is not possible in cases when the woman is in poor health or the disease is advanced.

Current screening techniques are challenged due to cost-ineffectiveness, variable false-positive results, and the asymptomatic nature of the early stages of ovarian cancer. Thus, it is required to develop an accurate, quick, convenient, and inexpensive method for detecting early cancer of ovaries at molecular level. Spectroscopy is increasingly used now days to characterize physical and chemical changes occurring in tissues and cells. It offers possibilities for new diagnostic and therapeutic approaches [7]. Spectroscopic techniques such as fluorescence and nuclear magnetic resonance (NMR) have been employed to distinguish cancerous and non-cancerous states of a tissue [8]. Fluorescence spectroscopy can provide biochemical information about the state of a tissue, but suffers from broad band fluorescence features [9]. There are only a small number of endogenous fluorophores in cancerous tissue to provide fluorescent signals and hence give rise to undesirable broad spectral features [10]. Tissue analysis by NMR spectroscopy requires highly sophisticated instrumentation and still suffers with unresolved peaks due to constrained molecular motions [11].

With the advances in vibrational spectroscopic techniques, its application in medical biology is increasing day by day [12,13]. Fourier transform infrared spectroscopy (FTIR) is a relatively simple, rapid and nondestructive technique that is adaptable for solids, liquids, and gases with a minimal sample preparation and can be used for both qualitative identification and the quantitative analysis of various components in a complex mixture [14,15]. Analysis of characteristic group frequencies in a spectrum allows qualitative estimates of chemical composition in these materials. Biomolecular features like conformational state, side chain length and inter/intra chain bondings can be measured easily using infrared spectroscopy. Recently, the application of infrared spectroscopy in biomedical sciences has increased a lot and various new clinical applications have been reported in the literature these applications include analysis of bone [16], skin [17], lung [18], breast [19], prostate [12] and cervical tissues [15]. Furthermore, this technique has been used in anticancer drug investigations [20-22], cancer grading (14), and studies on nucleic acid from tumor cells [23]. Fourier transform infrared spectroscopy has been extensively employed in the field of cancer research to address the problems of tumor biology [24-30]. The results of our previous research have shown its advantage in discrimination of breast cancer tissue from normal breast tissue [31]. In the present work, we examine the cancerous and normal tissues of ovaries to obtain information about ovarian cancer at molecular level with FTIR technique.

## Methods

### Tissue sampling

Tissue samples of 12 cases of ovarian cancer were obtained from Dharamshila Hospital, Delhi. Informed consent from patients have been taken prior to surgery. Post surgical cancer tissue and normal tissue (2-3 cm away from the tumor) samples were collected. All the samples were of stage II and III. For each case two samples were cut, one was put on the glass slide and was used for histological review. The other part of the tissue was frozen (-28°C) to obtain cryostat sections (2-4 μm) which were taken on zinc selenide (ZnSe) crystal plates. The tissue sections were placed on the ZnSe plates without any fixative and were used for spectral analysis.

### Spectral measurements

Varian 660 IR spectrometer equipped with DTGS detector and KBr beam splitter was used to record the spectra. FTIR spectra were collected in the transmission mode. The spectra were scanned in the mid-IR range from 650 to 4000 cm^-1 ^with a resolution of 4 cm^-1^. Two hundred and fifty six scans were collected for each spectrum and the spectra were ratioed against the background spectrum. The spectra were normalized after the baseline correction. Second order derivative of all the spectra were calculated using savitzky-Golay 2^nd ^order polynomial with 11 data points.

## Results and discussion

**Figure 1:**
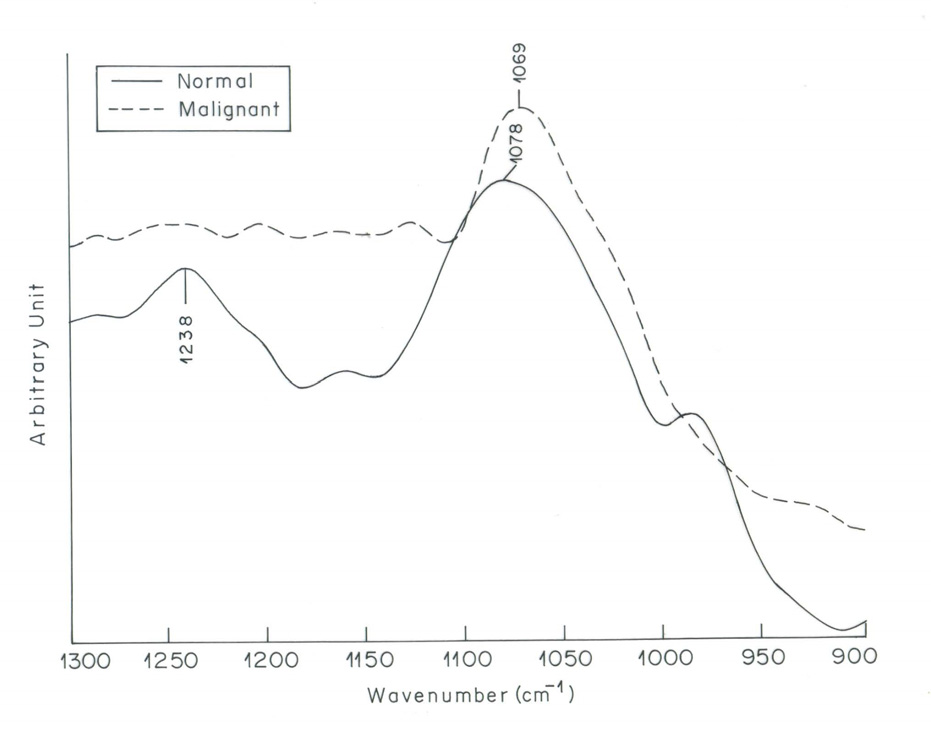
**Overlaid IR spectra of normal and malignant ovarian tissue in the region 900-1300 cm^-1^**.

**Figure 2:**
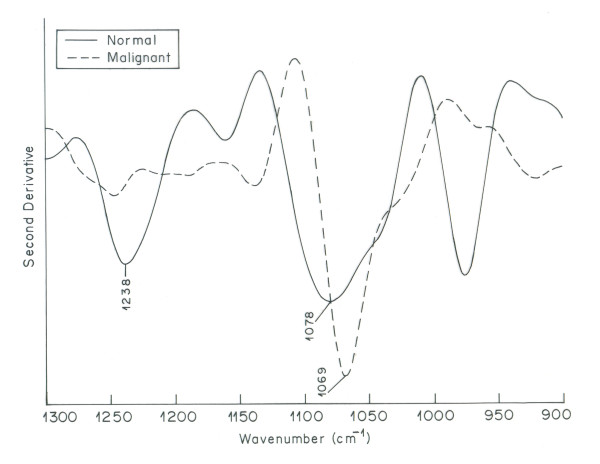
**Overlaid second order derivative IR spectra of normal and malignant ovarian tissue in the region 900-1300 cm^-1^**.

The spectra of the normal and cancerous ovarian tissue from different patients were recorded. The infrared spectrum of ovarian cancer tissue was found to be different from infrared spectrum of normal ovarian tissue. The malignant tissue exhibited deviations, in infrared bands assigned to biomolecular bonds, from their normal counterparts in all the cases studied. The malignant ovarian tissue spectra appeared to be more complicated as compared to normal ovarian tissue spectra. The spectral assignments were based on literature [29]. Figure 1 shows the overlaid IR spectra of the normal and malignant tissue in the region 900-1300 cm^-1 ^[32] The two major bands in this region at 1078 cm^-1 ^and 1238 cm^-1 ^are mainly due to the symmetric and asymmetric stretching modes of phosphodiester groups respectively [15,33]. As most of the phosphodiester groups in biological tissues are found in nucleic acids [34,35], these two bands are associated to the nucleic acid content of a cell. Malignant tissue shows a strong peak at 1069 cm^-1^, which is present as a broad peak of lesser intensity at 1078 cm^-1 ^in the spectrum of normal tissue. The anti symmetric phosphate stretching vibrations at 1238 cm^-1 ^in normal tissue appears as a broad shoulder in the spectrum of malignant tissue. The spectral shifting and increased intensity of phosphate bands become clearer in the second order derivative spectra of the region 900-1300 cm^-1^(Figure 2). The difference observed for symmetric and anti symmetric phosphate vibrations indicate towards the higher content of DNA in malignant tissue caused by characteristic endless replication of DNA in cancerous cells. The results obtained for nucleic acid are in corroboration with the findings of Anastassopoulou et al and Krafft et al, where increased intensity of nucleic acid bands were observed in cancerous tissue suggesting higher proliferative activity in malignant cells compared to the normal ones [36,37].

**Figure 3:**
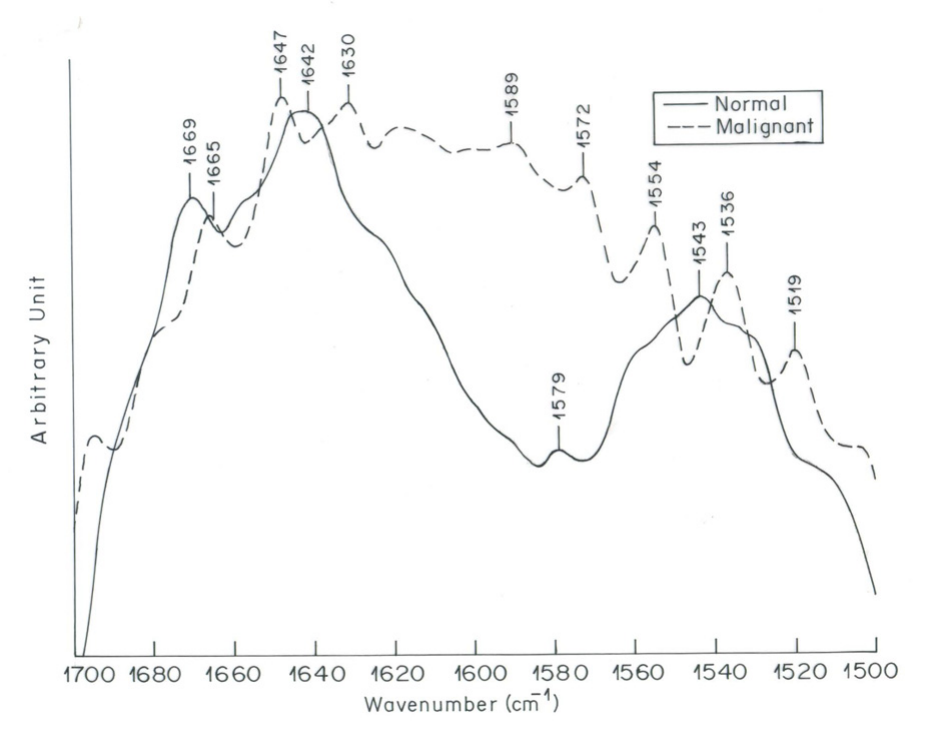
**Overlaid IR spectra of normal and malignant ovarian tissue in the region1500-1700 cm^-1^**.

**Figure 4:**
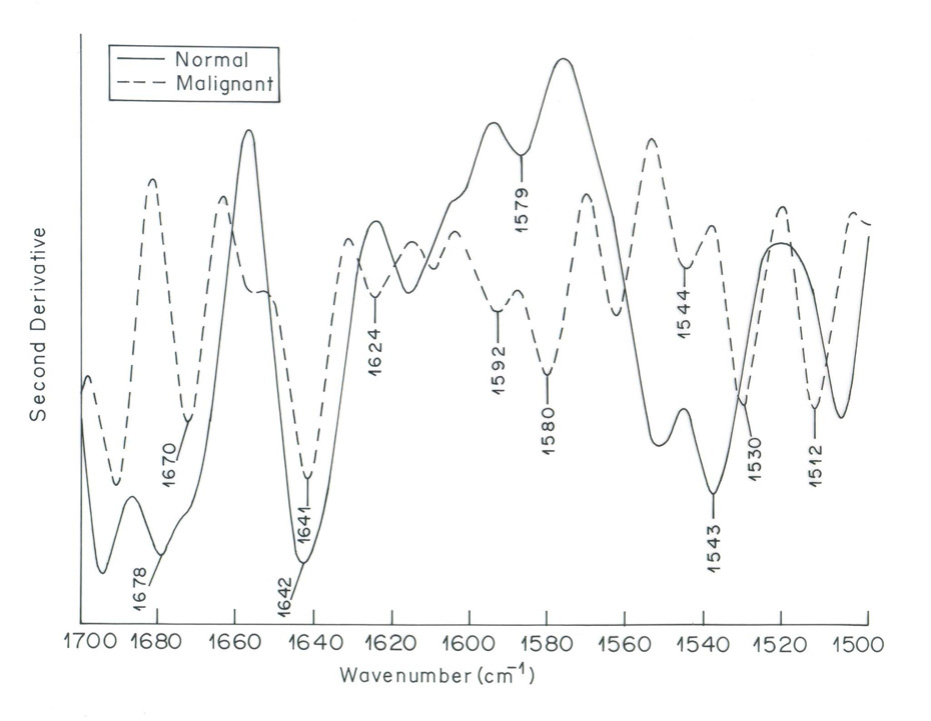
**Overlaid second order derivative IR spectra of normal and malignant ovarian tissue in the region 1500-1700 cm^-1^**.

Significant difference between the normal and malignant ovarian tissue spectra is observed in the region of 1500-1700 cm^-1 ^(Figure 3). This region denotes amide I, II and III bands of proteins. Vibrational bands at 1630, 1642 and 1647 cm ^-1 ^(amide I) arise mainly due to C = O stretching vibrations of the amide group of the protein backbone. These are primarily characterized by the alpha helix secondary structure of proteins [38]. The absorption bands at 1536, 1543 and 1554 cm^-1 ^arising from amide N-H bending vibrations are attributed to beta sheet secondary structure of proteins [39-41]. This spectral region is sensitive to changes in the molecular geometry and hydrogen bondings of peptide groups [39]. In comparison to normal tissue, malignant tissue spectrum exhibits shifting along with intensity variation in the bands assigned to alpha and beta structures. The increase in intensity is more prominent in the region assigned to beta structure as compared to alpha structure in the spectrum of malignant tissue. This could be attributed to alpha to beta conversion in the secondary structure of proteins in malignant tissue. These results are in corroboration with the findings of Yamada et al where the analysis of secondary structure of proteins reveal increased amount of beta sheet in necrotic area of carcinoma as compared to alpha helix [24]. Moreover the bands in the protein region are disturbed in the spectrum of malignant tissue as compared to clear IR bands in the spectrum of normal ovarian tissue. Second order derivative spectra of protein (Figure 4) region clearly depicts that IR bands of proteins in the malignant tissue are complicated and more in number as compared to normal tissue. Proteins play an important role in the physiological processes of living systems. Major functions of an organism are regulated by enzymes and hormones which are proteins. Protein content of a cell can be considered a diagnostic tool to determine the physiological phase of a cell [42]. The depletion of protein profile in the spectrum of malignant ovarian tissue indicates towards induced diversification of energy to meet the impending energy demands during the malignant stress of cell [43].

**Figure 5:**
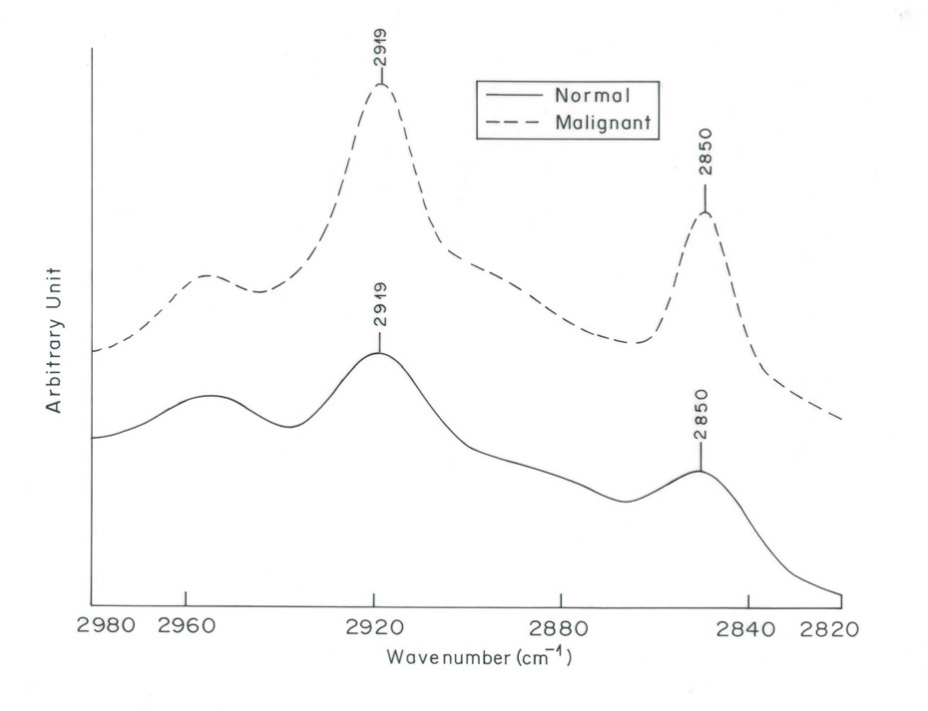
**Overlaid IR spectra of normal and malignant ovarian tissue in the region 2820-2890 cm^-1^**.

**Figure 6:**
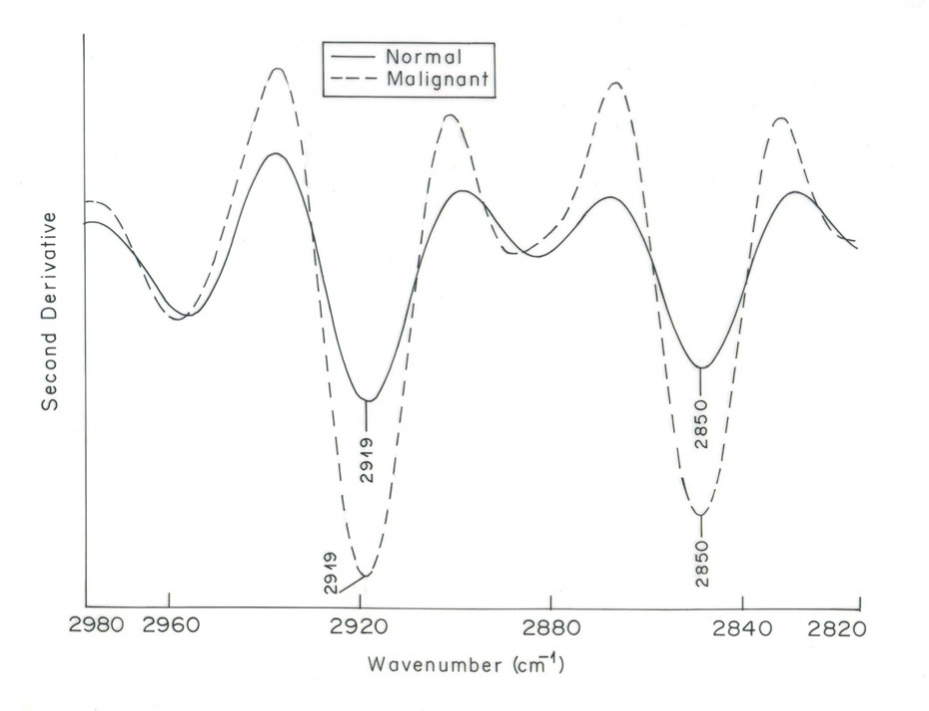
**Overlaid second order derivative IR spectra of normal and malignant ovarian tissue in the region 2820-2890 cm^-1^**.

Figure 5 shows the overlaid IR spectra of normal and malignant tissue in the region 2820 - 2980 cm^-1^. This region is associated with the stretching vibrations of lipid hydrocarbons. Remarkable changes are observed in this region for malignant tissue as compared to its normal counterpart. Two peaks at 2850 cm^-1 ^and 2919 cm^-1 ^result from stretching vibrations of the CH_2 _and CH_3 _groups in acyl chains of lipids [38]. These peaks underwent a significant increase in intensity in malignant tissue as compared to normal tissue. The increase in intensity is more clearly seen in second order derivative spectra of normal and malignant tissue in the region 2820-2980 cm^-1^(Figure 6). This increase in intensity indicates enhancement in lipid contents in malignant cells. These results are in corroboration with the findings of struchkov et al where considerable increase of neutral lipids in nulei of Ehrlich ascites carcinoma was observed [44]. Also tumor cells have dysregulated metabolism as compared to normal cells; they undergo glycolytic rather oxidative metabolism and synthesize greater amount of fatty acids than normal cells. It is also reported earlier that tumor cells exhibit increase in de novo fatty acid synthesis, where as normal cells are thought to acquire fatty acids primarily from dietary sources [45]. Nomura et al demonstrate the increase of an enzyme monoacyl glycerol lipase (MAGL) in high grade human ovarian cells, due to which the lipid content of malignant cells increases [46]. These reports support our observation of increased intensity in the characteristic lipid bands in the IR spectrum of malignant ovarian tissue.

## Conclusion

The results of the present study have shown that remarkable difference exist between the IR spectra of normal and malignant tissue in terms of absorption frequencies and intensities of prominent absorption bands of cellular biomolecules. The differences observed in the spectra of normal and malignant tissue reflect changes in the content of nucleic acid and lipids. Protein absorption bands indicate the presence of new proteins as well as changes in their conformation and composition. Spectral absorption patterns observed for major biomolecules; nucleic acid, proteins and lipids can be viewed as IR spectral signatures which can be used for distinguishing malignant ovarian tissue from the normal tissue. Based on this, we can compare the infrared spectrum of malignant tissue with its corresponding normal tissue, and establish a new way to diagnose malignant tumors. Prospectively, in conjunction with other markers this technique could be useful in diagnosis of ovarian cancer.

## Competing interests

The authors declare that they have no competing interests.

## Authors' contributions

RM contributed in the conception and design of the idea, interpreted the data, performed the statistical analysis and given final approval for the version to be published. GT contributed towards acquisition and analysis of data and preparation of manuscript. DKJ participated in coordination of the study and helped to design the manuscript. RD and NG provided the samples, helped in biological corroboration of spectral data and revision of manuscript. All authors read and approved the final manuscript.
